# A Case of Atypical Acute Encephalopathy Unmasking IgA Multiple Myeloma

**DOI:** 10.7759/cureus.98689

**Published:** 2025-12-08

**Authors:** Jamie T Abad, Tasnim Rahman, Renieh Nabaty, Omar Abbas, Kunj Patel, Nicholas Sturla

**Affiliations:** 1 Internal Medicine, Henry Ford Health System, Detroit, USA; 2 Pathology and Laboratory Medicine, Henry Ford Health System, Detroit, USA; 3 Cardiology, Henry Ford Health System, Detroit, USA

**Keywords:** blood hyperviscosity, hyperammonemia encephalopathy, iga lambda multiple myeloma, multiple myeloma, myeloma-related encephalopathy

## Abstract

Multiple myeloma (MM) can have an insidious onset, particularly in its early stages, which may contribute to delays in diagnosis. Acute encephalopathy is a rare and atypical manifestation of MM that can further complicate timely recognition and management. We present the case of a 57-year-old woman who initially reported fatigue, back pain, recurrent falls, and significant unintentional weight loss and was found to have hypercalcemia, anemia, acute renal dysfunction, and bone lesions. Her clinical course rapidly evolved into profound encephalopathy, characterized by a change in mentation from her baseline, agitation, and eventual unresponsiveness requiring intubation.

Extensive investigation, including a bone biopsy, revealed elevated IgA lambda paraproteins consistent with MM. She also had borderline serum hyperviscosity, mildly elevated ammonia levels, and diffuse microhemorrhages on brain MRI. Despite interventions, including plasmapheresis and high-dose steroids, her encephalopathy persisted until initiation of bortezomib-based chemotherapy, resulting in neurological improvement. This case emphasizes the importance of recognizing MM as a potential cause of unexplained encephalopathy and highlights the complexity of its neurological manifestations, underscoring the need for prompt hematologic evaluation and interdisciplinary management.

## Introduction

Multiple myeloma (MM) is a clonal plasma cell proliferative disorder characterized by an abnormal increase in monoclonal immunoglobulins or paraproteins. MM is predominantly seen in the geriatric population, with a median age at diagnosis of 69 years [1]. The accumulation of malignant plasma cells in the bone marrow can lead to specific end-organ damage. Most commonly, clinical manifestations include hypercalcemia, renal dysfunction, anemia, or bone pain accompanied by lytic lesions, summarized by the “CRAB” criteria (hypercalcemia, renal dysfunction, anemia, and bone lesions). While the classic CRAB criteria are well recognized, neurological manifestations are less commonly encountered and often present diagnostic and therapeutic challenges. These complications are diverse, ranging from peripheral neuropathy to rarer but serious conditions such as CNS involvement and cerebrovascular events [2].

Encephalopathy is particularly important to recognize. It encompasses a wide range of potential etiologies. In the context of MM, it can be a clinically significant neurological complication arising from multiple mechanisms, including metabolic derangements (such as hypercalcemia, uremia, or hyperviscosity), paraneoplastic syndromes, drug toxicity, or direct CNS involvement. Typically, acute encephalopathy presents with global cognitive dysfunction or altered consciousness and can be evaluated through laboratory testing, neuroimaging, and cerebrospinal fluid (CSF) analysis.

Although the precise pathophysiology of MM-induced encephalopathy remains unclear, it can be attributed to the effects of abnormal proteins produced by myeloma cells, leading to neurotoxicity. Hyperviscosity syndrome occurs in approximately 2-6% of MM cases, more commonly in patients with elevated IgA or IgM paraproteins due to their molecular structure and polymerization behavior [3]. Hyperammonemic encephalopathy, another rare but recognized metabolic complication of MM, has been reported even in the absence of liver disease and is hypothesized to result from amino acid metabolism by myeloma cells [4]. A large pooled analysis identified 40 cases of MM-induced hyperammonemic encephalopathy, indicating a relatively low incidence [5]. Hyperammonemic encephalopathy is particularly devastating, with an in-hospital mortality rate approaching 48% and universal mortality reported in patients not treated with myeloma-directed therapy [5]. Although hyperammonemia and hyperviscosity have been described, their co-occurrence is exceedingly rare, with only a few cases reported in the literature, including a series by Kuribayashi et al. describing three patients with MM or plasma cell leukemia who developed both complications and improved with chemotherapy [6]. Early recognition and prompt management, typically involving treatment of the underlying MM, is crucial for improving neurological outcomes.

We report the case of a previously healthy woman who presented with recurrent falls and later developed profound encephalopathy and was found to have IgA lambda MM. This case highlights the diagnostic complexity and the importance of considering hematologic malignancy in the differential diagnosis of unexplained encephalopathy, particularly in the presence of subtle systemic clues.

## Case presentation

A 57-year-old woman with a past medical history significant for hypertension, type 2 diabetes mellitus, and osteoporosis initially presented to her primary care physician with progressive mid-back pain and right rib pain following multiple minor traumatic events. These included two mechanical falls over the past few months and an episode of acute back strain after lifting a heavy object the month prior. She initially pursued conservative management with chiropractic care and massage therapy, but persistent pain prompted further evaluation. A computed tomography (CT) scan noted an anterior compression fracture of the T8 vertebral body. Subsequent magnetic resonance imaging (MRI) noted chronic compression fractures at T8 and T10, each with approximately 50% vertebral height loss. The T8 deformity showed slightly increased central height loss and minimal edema without significant spinal canal stenosis or nerve root compression. She was evaluated for bracing, initiated on conservative management, and was scheduled for follow-up with neurosurgery to determine the potential need for a kyphoplasty. While these imaging studies informed clinical management, the original image files were not accessible for inclusion in this report.

Over the following weeks, she developed worsening fatigue, back pain, weakness, recurrent falls, and unintentional weight loss. These concerning systemic symptoms prompted her presentation to the emergency department. On physical examination, the patient was alert but appeared fatigued. She was oriented to person, place, and time; however, she was somewhat slow to respond to questions. Cranial nerves II-XII were intact. Motor exam revealed 3/5 strength in all extremities with symmetric proximal and distal involvement. Reflexes were symmetric, and the passive range of motion was preserved. Sensation to touch and pinprick was intact throughout. Cerebellar testing was limited by weakness. No joint swelling or tenderness was noted. There was no lymphadenopathy or hepatosplenomegaly on exam.

Initial laboratory workup was notable for normocytic anemia, acute kidney injury, hypercalcemia, and mild transaminitis (Table 1). 

**Table 1:** Initial laboratory workup MCV, mean corpuscular volume; AST, aspartate aminotransferase; ALT, alanine aminotransferase

Test	Result	Reference Range
White blood cell count	6.4 K/uL	3.8-10.6 K/uL
Hemoglobin	6.6 g/dL	12.0-15.0 g/dL
MCV	98.1 fL	80-100 fL
Platelets	215 K/uL	150-450 K/uL
Creatinine	3.12 mg/dL	<1.16 mg/dL
Blood urea nitrogen	43 mg/dL	10-25 mg/dL
Bicarbonate	15 mmol/L	21-35 mmol/L
Anion gap	21	3-13
Calcium	15.4 mg/dL	8.2-10.2 mg/dL
AST	67 IU/L	<35 IU/L
ALT	34 IU/L	<52 IU/L

**Figure 1:**
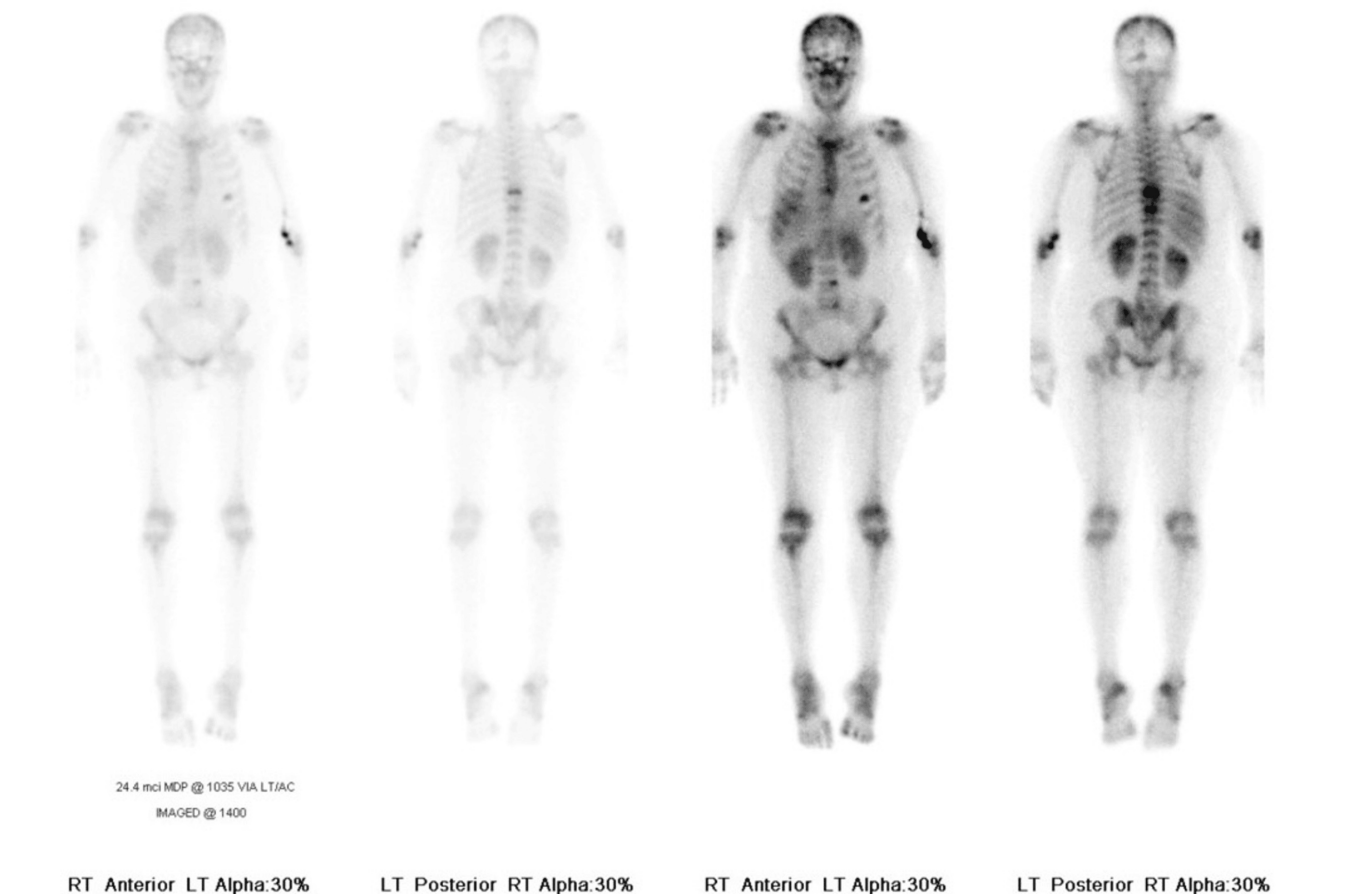
Whole-body nuclear imaging showing focal skeletal abnormalities​ Nuclear imaging demonstrates focal radiotracer uptake at the known compression deformities of the T8 and T10 vertebrae. Additional uptake is seen in the anterior left fifth rib, suggestive of interval post-traumatic change.

**Figure 2:**
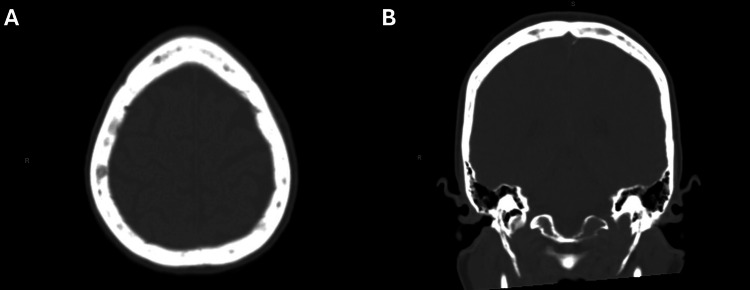
CT of the head - axial and coronal views A (axial view) and B (coronal view) demonstrate multifocal, nonspecific osseous lucencies within the right supraorbital rim and calvarium, concerning for osseous metastases or myelomatous involvement. Additional findings include age-related cerebral volume loss and mild paranasal sinus disease. CT, computed tomography

Whole-body nuclear imaging revealed focal radiotracer uptake at T8 and T10 vertebral bodies and the anterior left fifth rib (Figure 1). CT of the head noted multifocal lucencies in the calvarium, including the right supraorbital rim and scattered lucencies over the bilateral convexities, concerning for osseous metastases or MM (Figure 2). She was admitted for management of hypercalcemia. Given the constellation of hypercalcemia, renal dysfunction, anemia, and bony involvement, there was high clinical suspicion for MM, and a diagnostic workup was initiated accordingly.

**Figure 3:**
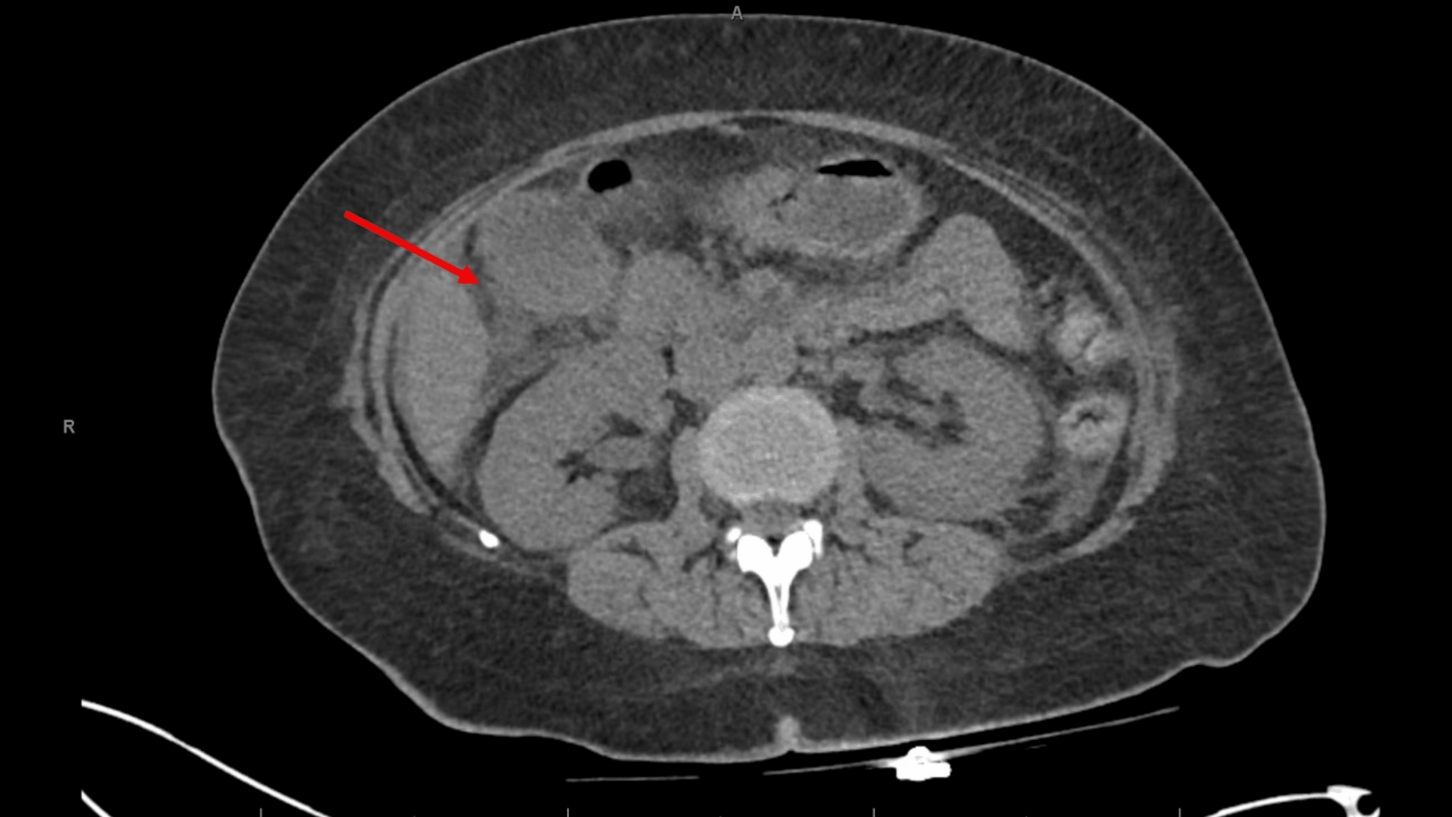
CT of the abdomen and pelvis CT imaging demonstrates gallbladder wall thickening with pericholecystic inflammatory stranding and fluid, findings concerning for acute cholecystitis. Small-volume ascites is also noted. CT, computed tomography

She received intravenous fluids, calcitonin, and pamidronate. Her serum calcium decreased from 13.2 mg/dL to 9.4 mg/dL within days. Shortly thereafter, her aspartate aminotransferase (AST) and alanine aminotransferase (ALT) acutely rose to 2,570 IU/L and 1,038 IU/L, respectively. Alkaline phosphatase remained relatively normal at 66 IU/L. She was empirically treated with N-acetylcysteine while pending further workup. Infectious and hepatic workup, including hepatitis serologies and acetaminophen levels, was unremarkable. Lactate was elevated at 4 mmol/L, and ammonia was elevated at 87 µmol/L (reference range 18-50 µmol/L). CT of the abdomen and pelvis showed gallbladder wall thickening with pericholecystic inflammatory stranding and fluid concerning for acute cholecystitis (Figure 3), although a subsequent hepatobiliary iminodiacetic acid scan showed no evidence of cystic duct obstruction or acute cholecystitis. Despite extensive evaluation, the etiology of her severe transaminitis remained unclear. The pattern favored a transient hepatocellular injury, with ischemic hepatopathy considered most likely, although no definitive cause was identified.

Concurrently, her mental status began to decline. Broad-spectrum antibiotics were initiated for empiric coverage. Blood and respiratory cultures were obtained and resulted positive for Streptococcus pneumoniae. Her mental status continued to decline. She became very lethargic, requiring intubation for airway protection. Another CT of the head was obtained, which showed known multiple destructive lytic lesions throughout the calvarium; however, there were no acute findings. Ammonia peaked at 98 µmol/L. She received lactulose for presumed hyperammonemia-induced encephalopathy; however, despite normalization of ammonia to 32 µmol/L, her mentation did not improve. EEG showed diffuse slowing without epileptiform activity. Brain MRI demonstrated known lesions within the calvarium and skull base, as well as numerous chronic microhemorrhages at the peripheral gray-white matter junction, but no acute infarct. She was empirically treated for meningitis with 14 days of antibiotics, including vancomycin, meropenem, ampicillin, and acyclovir. Lumbar puncture with CSF studies was notable for elevated lactate (Table 2), but the remainder of the CSF studies and infectious workup were unremarkable.

**Table 2:** Summary of CSF analysis Paraneoplastic panel: All CSF autoantibodies tested, including amphiphysin, AGNA-1, ANNA-1/2/3, CRMP-5-IgG, PCA-Tr, PCA-1, and PCA-2, were negative. VZV, Varicella zoster virus; VDRL, venereal disease research laboratory test; HSV, herpes simplex virus; NMDA: N-methyl-D-aspartate receptor Ab IgG; CSF, cerebrospinal fluid

Test	Result	Reference Range
Color	Colorless	Colorless
Clarity	Clear	Clear
Glucose	104 mg/dL	40-80 mg/dL
Protein	34.2 mg/dL	15-55 mg/dL
Red blood cell	2	0/cu mm
Total nucleated cell count	2	0-5/cu mm
Neutrophils	0	0-6%
Basophils	0	0%
Eosinophils	0	0%
Macrophages	0	0%
Lymphocytes	67%	40-80%
Monocytes	0	0%
Lactic acid	3.1 mmol/L	1.2-2.4 mmol/L
VZV	Not detected	Not detected
VDRL	Nonreactive	Nonreactive
HSV1/2 DNA	Not detected	Not detected
EBV	Not detected	Not detected
West Nile IgG and IgM	Negative	Negative, <1.30
Lyme Borrelia burgdorferi antibodies	0.00	≤0.99 LIV
NMDA receptor	<1:1	<1:1
Oligoclonal bands	Negative	Negative
Cryptococcal antigen	Negative	Negative
Toxoplasma	Negative	Negative
14-3-3 protein (Creutzfeldt-Jakob disease)	Negative	<30-1,999 AU/mL
Histoplasma antigen	None detected	None detected
Cryptococcal antigen	Negative	Negative
Blastomyces antigen	None detected	None detected
T-tau protein	213	0-1,149 pg/mL
Paraneoplastic panel	Negative	Negative
Cytology	Negative	Negative

**Figure 4:**
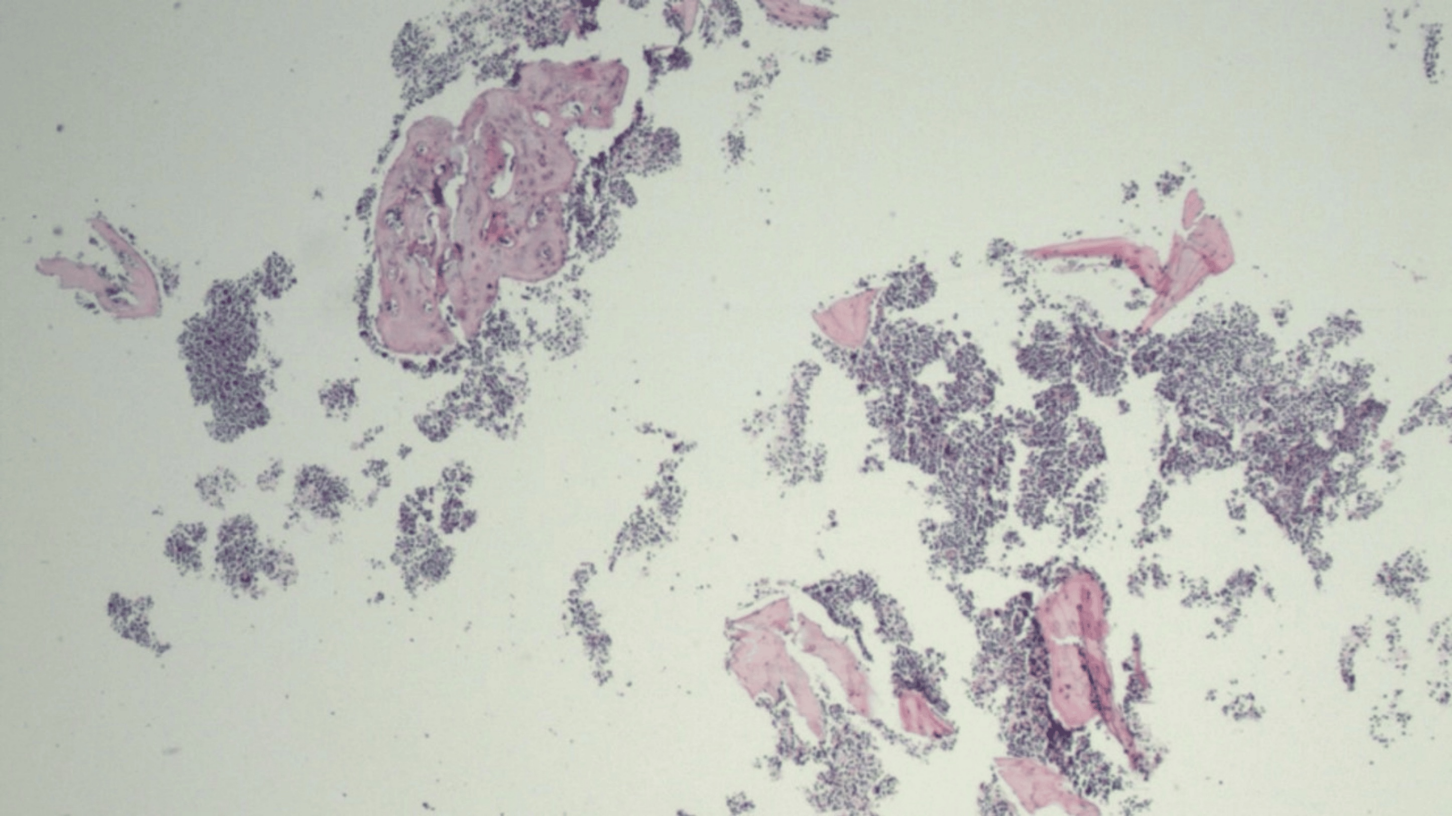
H&E stain of T10 bone marrow biopsy (40x magnification) Needle-core biopsy of the T10 vertebral body demonstrates minute fragments of bone with intertrabecular clusters of plasma cells amid a background of trilineage hematopoiesis. Findings are consistent with a plasma cell neoplasm. H&E, hematoxylin and eosin

**Figure 5:**
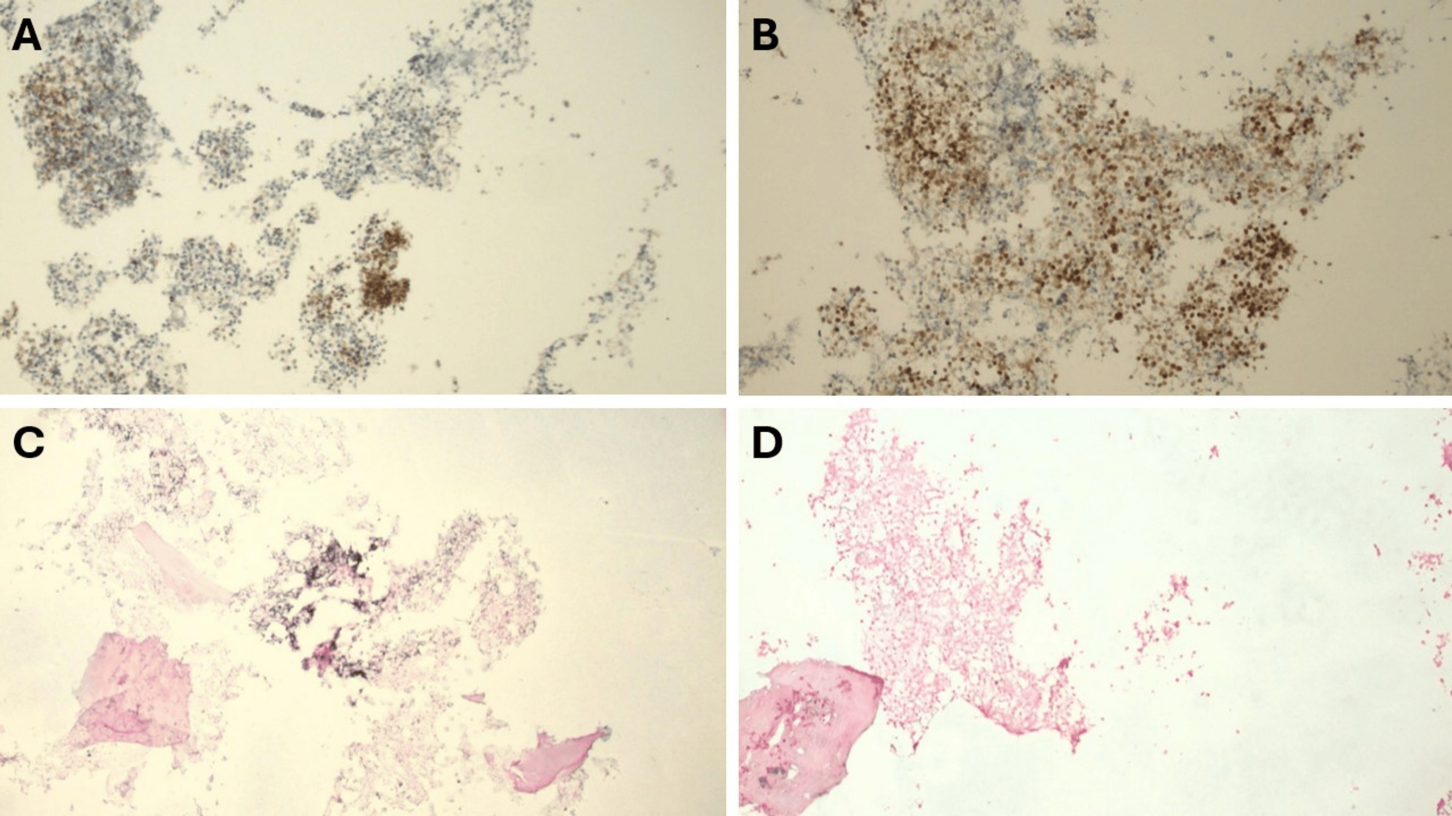
Immunohistochemical and in situ hybridization stains of T10 bone marrow biopsy (100x magnification) (A) CD138 immunohistochemical stain highlights clusters of plasma cells. (B) MUM1 immunohistochemical stain similarly demonstrates plasma cell clusters. (C) Lambda in situ hybridization shows monotypic plasma cells with lambda light chain restriction. (D) Kappa in situ hybridization demonstrates minimal staining, supporting lambda light chain restriction and confirming clonality.

Serum electrophoresis showed that serum IgA was markedly elevated at 5,525 mg/dL (reference range 70-400 mg/dL), total protein was 8.6 g/dL (reference range 6.6-8.5 g/dL), and beta globulins were 3.47 g/dL (reference range 0.7-1.29 g/dL). M-protein quantification revealed two monoclonal IgA lambda components measuring <2.5 g/dL and 0.4 g/dL, respectively, with associated suppression of polyclonal gammaglobulins and hypoalbuminemia. Additional laboratory workup was notable for borderline elevated plasma viscosity at 1.91 cP (reference range 1.5-1.9). Needle core bone biopsy was consistent with a plasma cell neoplasm (Figure 4 and Figure 5).

The encephalopathy was presumed to be multifactorial in etiology, potentially due to systemic infection, CNS involvement by myeloma, and metabolic dysfunction. Neurology suspected MM-associated hyperviscosity as a contributor to cerebral hypoperfusion, supported by the elevated lactate levels in the CSF.

She was trialed on dexamethasone (40 mg daily x 4 days) without clinical improvement. Given concern for hyperviscosity syndrome, she underwent three sessions of therapeutic plasma exchange (PLEX), after which serum IgA levels decreased from 5,525 mg/dL to 3,025 mg/dL and then to 2,739 mg/dL. Despite this serological improvement, her encephalopathy persisted. Hematology recommended initiation of systemic therapy with bortezomib and dexamethasone. Roughly one week after her first cycle of chemotherapy, she demonstrated significant clinical improvement, becoming more alert, verbal, and able to follow commands. She completed three cycles of bortezomib and dexamethasone while inpatient, as plans were being made for discharge to a skilled nursing facility.

## Discussion

While the CRAB criteria are the hallmark of myeloma-related end-organ damage, neurologic manifestations such as altered mental status remain diagnostically and therapeutically challenging. Neurologic complications are not typically part of the initial presentation of MM, but when they occur, they can reflect advanced disease or atypical biology [1]. Common metabolic etiologies include hypercalcemia, uremia secondary to renal impairment, hyperammonemia, and hyperviscosity syndrome. CNS involvement should also be considered, including direct plasma cell infiltration leading to meningeal involvement, cerebral edema, or intraparenchymal lesions. Because of the abnormal proliferation of malignant plasma cells and suppression of normal immunoglobulin production, patients with MM are immunocompromised and thus susceptible to infectious causes of altered mental status.

In our case, an extensive diagnostic workup was pursued, including brain imaging, EEG, lumbar puncture with CSF analysis, and paraneoplastic panel testing, all of which were largely unremarkable. Infectious and hepatic causes were ruled out. Brain MRI demonstrated diffuse chronic microhemorrhages, and CSF analysis revealed elevated lactate, findings that raised concern for subacute cerebral hypoperfusion in the context of hyperviscosity syndrome.

Although the patient’s measured plasma viscosity was only borderline elevated (1.91 cP), she had markedly elevated IgA levels (5,525 mg/dL), and the dimeric nature of IgA makes it more likely to cause hyperviscosity at lower concentrations than IgG [3]. Although her viscosity value was below the traditional threshold for clinical concern, symptoms of hyperviscosity can occur at lower levels in IgA myeloma due to increased molecular size and polymerization. Hyperviscosity may impair perfusion and exacerbate toxin accumulation, potentially contributing to neurologic dysfunction [7]. Therapeutic plasma exchange was performed, resulting in a significant reduction in IgA levels, but her mental status did not improve, suggesting a multifactorial process or irreversible neurologic injury. Hyperviscosity syndrome is an under-recognized but clinically significant complication in patients with MM, particularly the IgA subtype, and may impair cerebral perfusion by causing sludging in small vessels, leading to ischemia or microvascular injury. This hypothesis was supported by the diffuse microbleeds seen on MRI and elevated lactate in the CSF, which can serve as a surrogate marker of cerebral hypoxia or low perfusion [3].

Concomitantly, hyperammonemia was considered a contributing etiology. While her ammonia levels peaked at 98 µmol/L, only modestly elevated, there are documented cases of MM-associated hyperammonemic encephalopathy even in the absence of hepatic dysfunction, particularly in patients with relapsed or high-burden disease [4,8]. Our patient had newly diagnosed MM with high tumor burden and markedly elevated IgA, possibly supporting this mechanism. The pathophysiology remains incompletely understood but may include excessive protein synthesis and catabolism by malignant plasma cells, impaired urea cycle activity, and rare hepatic infiltration or portosystemic shunting [9]. Reports suggest that ammonia can be generated by myeloma cells in the absence of liver dysfunction, contributing to encephalopathy and poor outcomes if not addressed with disease-directed therapy [10,11]. Although her liver enzymes normalized and imaging showed no hepatic involvement, the elevation in ammonia likely contributed to her encephalopathy, and the lack of response to lactulose suggested that myeloma-directed therapy was necessary to reverse the metabolic insult. While both hyperammonemia and hyperviscosity are individually recognized complications, their co-occurrence is rare and sparsely reported. A case series described three patients with plasma cell disorders who experienced both, all of whom improved with chemotherapy [6]. 

Other rare causes of encephalopathy, such as paraneoplastic autoimmune encephalitis and drug-induced syndromes like PRES, were considered but were less likely in this case. Extensive CSF studies, including paraneoplastic panels and infectious workup, were negative. Posterior reversible encephalopathy syndrome (PRES) has been reported with bortezomib and other chemotherapeutic agents, but the timing and imaging findings did not support this etiology [12]. The absence of seizures, visual changes, and vasogenic edema on MRI further reduced the likelihood of PRES. Nonetheless, these entities remain important differentials, especially in patients who have undergone stem cell transplantation or received neurotoxic agents, although they were not consistent with our patient.

The patient’s encephalopathy was refractory to initial therapies, including corticosteroids, lactulose, and plasma exchange. Approximately one week following her first cycle of systemic chemotherapy with bortezomib and dexamethasone, she exhibited significant neurologic improvement. This improvement occurred in parallel with a marked decline in serum IgA levels, reinforcing the link between disease burden and neurologic dysfunction. It supports the hypothesis that her encephalopathy was primarily related to her underlying myeloma, whether due to metabolic effects, hyperviscosity, or both. Previous case reports have similarly documented neurologic recovery following myeloma-directed therapy, underscoring the importance of prompt recognition and intervention [9,12].

## Conclusions

This case illustrates the importance of maintaining a broad differential for acute encephalopathy and underscores that MM, particularly the IgA subtype, can present with neurologic symptoms driven by hyperviscosity or metabolic derangements. Early recognition and initiation of myeloma-directed therapy may be essential for neurologic recovery. An interdisciplinary approach that includes neurology, hematology/oncology, and critical care is often necessary to guide diagnosis and management in these complex cases.
